# Constructing two-level nonlinear mixed-effects crown width models for Moso bamboo in China

**DOI:** 10.3389/fpls.2023.1139448

**Published:** 2023-02-16

**Authors:** Xiao Zhou, Zhen Li, Liyang Liu, Ram P. Sharma, Fengying Guan, Shaohui Fan

**Affiliations:** ^1^ International Center for Bamboo and Rattan, Key Laboratory of National Forestry and Grassland Administration, Beijing, China; ^2^ National Location Observation and Research Station of the Bamboo Forest Ecosystem in Yixing, National Forestry and Grassland Administration, Yixing, China; ^3^ Institute of Forestry, Tribhuwan University, Kritipur, Kathmandu, Nepal

**Keywords:** growth function, random effect, variance-stabilizing function, sampling strategy, bamboo forest management

## Abstract

Bamboo crown width (CW) is a reliable index for evaluating growth, yield, health and vitality of bamboo, and light capture ability and carbon fixation efficiency of bamboo forests. Based on statistical results produced from fitting the eight basic growth functions using data from 1374 *Phyllostachys pubescens* in Yixing, Jiangsu Province, China, this study identified the most suitable function (logistic function) to construct a two-level mixed effects (NLME) CW model with the forest block and sample plot-level effects included as random effects in the model. Four methods for selecting sample bamboos per sample plot (largest bamboo, medium-sized bamboo, smallest bamboo, and randomly selected bamboos) and eight sample sizes (1–8 selected bamboos per sample plot) were evaluated to calibrate our NLME CW model. Using diameter at breast height (DBH), height to crown base (HCB), arithmetic mean diameter at breast height (MDBH), and height (H) as predictor variables, the model produced the best fit statistics (Max R^2^, min RMSE, and TRE). This model was further improved by introducing random effects at two levels. The results showed a positive correlation of CW with HCB and DBH and a negative correlation with H. The smallest two bamboo poles per sample plot used to estimate the random effects of the NLME model provided a satisfactory compromise regarding measurement cost, model efficiency, and prediction accuracy. The presented NLME CW model may guide effective management and carbon estimation of bamboo forests.

## 1 Introduction

Tree crowns represent a crucial site for material exchanges and energy conversions between forests and the environment (Fu et al., 2017a; Fu et al., 2017b; Lei et al., 2018; Pan et al., 2020). Variables related to tree crowns are important predictors of forestry models, including those for crown length (Tahvanainen and Forss, 2008; Fu et al., 2017a; Fu et al., 2017b; Fu et al., 2017c), crown length to tree height ratio (Tahvanainen and Forss, 2008; Leites et al., 2009; Fu et al., 2015), height to crown base (HCB) (Fu et al., 2017c; Yang et al., 2020; Pan et al., 2020; Zhou et al., 2022), and crown width (CW) (Bragg, 2001; Sánchez-González et al., 2007; Stephanie and Stephen, 2011; Ma et al., 2022). CW, which is half of the sum of crown radii and is measured in four directions of south, east, north, and west (Fu et al., 2017b; Fu et al., 2013; Paulo et al., 2015; Fu et al., 2017a; Lei et al., 2018) reflects the vitality and competitiveness of plants. Analyzing CW and understanding its relations to other factors will enable predicting crown productivity (Larocque and Marshall, 1994), forest mortality (Monserud and Sterba, 1996), and forest biomass (Horntvedt, 1993; Hoffmann and Usoltsev, 2002). Crown diameter is also an important parameter for stand visualization (Lei et al., 2006). Detailed information on tree crowns can assist the management of ecosystem characteristics, such as forest productivity, biodiversity, and wildlife habitats.

Despite these benefits, measuring the CW of each tree in all sample plots is time-consuming, laborious, and costly (Bragg, 2001; Buchacher and Ledermann, 2020). CW measurements of representative sample trees per sample plot from an adequate number of sample plots allocated across forests are necessary to build high-precision CW models.

Early studies on applying the CW model used the diameter at breast height (DBH) as a predictor (Hetherington, 1967). Today, researchers prefer to add additional factors to the CW model to reduce potential bias. These factors may include tree size and stand vitality (e.g., stand age, stand density, canopy density, tree height, HCB), site quality (site index), stand competition factors (Fu and Sun, 2013; Fu et al., 2013; Ma et al., 2022) and other environmental factors such as slope, direction, and location (Zarnoch et al., 2004 ;Fu et al., 2017b; Fu et al., 2017c; Fu and Sun, 2013; Fu et al., 2013; Fu et al., 2017a; Lei et al., 2018).

CW data needed to build CW models are often acquired from varying growth conditions within different forest stands, which creates nested conditions. Thus, similar to other tree attribute data, CW data are more or less hierarchically structured, and observations are most likely correlated with each other. Using ordinary least squares (OLS) regression to estimate the CW models with the nested data structure will lead to a significant bias (West et al., 1984; Zhang et al., 2017). Mixed-effects modeling, which effectively addresses the aforementioned observation dependence [the data are hierarchically structured (a sample plot nested in the blocks)] problems, must be applied to reduce potential bias (Fu et al., 2013; Yang et al., 2020; Pan et al., 2020; Zhou et al., 2021a; Zhou et al., 2021b; Ma et al., 2022). Mixed-effects modeling accounts for most of the heterogeneity and randomness caused by known or unknown factors. Mixed-effects models demonstrate a promising flexibility because only the fixed effect part [M response model (without random effects)] or both fixed and random effects parts following response calibration can be applied (Fu et al., 2017c; Fu et al., 2013; Yang et al., 2020; Zhou et al., 2021a). Most CW models have been based on data from tree species (Fu and Sun, 2013; Fu et al., 2013; Fu et al., 2017c; Lei et al., 2018; Ma et al., 2022). However, bamboo CW modeling is limited.

Because of the global climate warming, the concentration of carbon dioxide in the atmosphere has reached the highest level of 410 ppm (Masson-Delmotte et al., 2021), carbon sequestration by growing forests is a cost-effective option for mitigating CO_2_ emission caused by human activities. Thus, the carbon cycle remains an important topic worldwide for research, and all green plants, including bamboo, play major roles for carbon storage. Although the world’s forest area has decreased continuously over the past 30 years, the planet’s bamboo forest area has increased by an average annual rate of 3% (FAO, 2010). Bamboo is an important carbon sink that can accumulate a large amount of carbon in a short period (Yen and Lee, 2011; Yen, 2016). Because bamboo forests are important in mitigating climate warming, the relationships between bamboo CW and various factors that influence CW should be investigated.

Based on existing research problems such as the absence of bamboo CW modeling, a hierarchical data structure, and correlations among the observations, we used *Phyllostachys edulis* (moso bamboo) as a target species in this study to (1) develop a two-level nonlinear mixed-effects (NLME) CW model (NLME model) through including multiple factors as predictor variables, (2) simulate the impacts of those factors on CW, and (3) select the optimal strategy to predict the random effects in the response calibration of the NLME CW model. The NLME CW model presented in this study can reduce the time and cost of CW measurements and help formulate bamboo forest management plans.

## 2 Materials and methods

### 2.1 Study area and data collection

**Figure 1:**
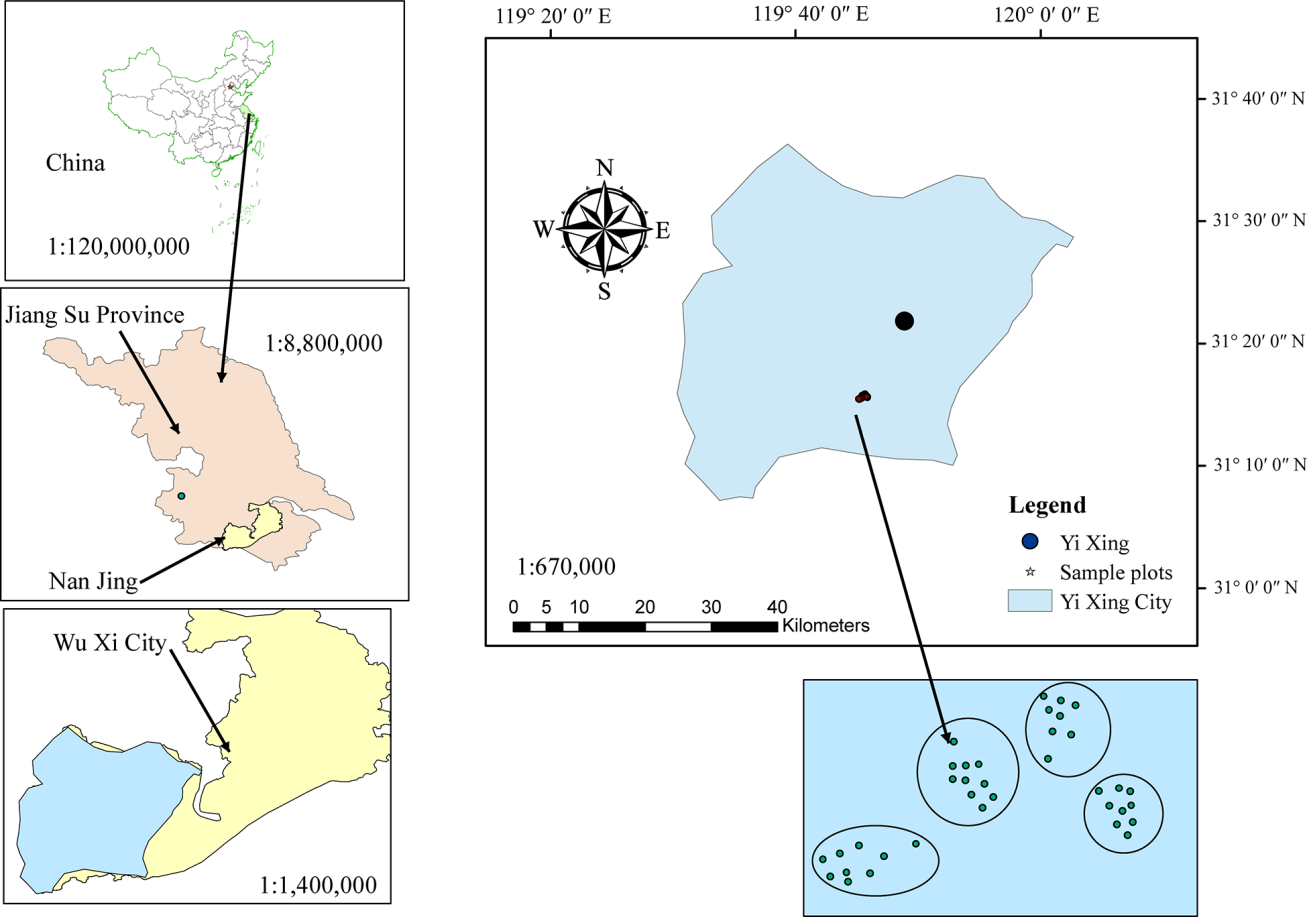
Location of the study area.

The study area is located in Yixing State, Forest Farm, Jiangsu Province (**Figure 1**). This transitional zone is a typical representation of the northern subtropical bioclimate, Jiangnan bamboo area, and scattered bamboo area. The forest farm has a subtropical monsoon climate, with high and low temperatures of 38.8 and -4.5 °C, respectively, an average annual temperature of 16.5 °C, and an average annual precipitation of 1229.9 mm. The main tree species on forest farms are *Cunninghamia lanceolata* (Lamb.) Hook., *Pinus massoniana* (Lamb.) var. *massoniana*, *Parrotia subaequalis* (H. T. Chang) R. M. Hao & H. T. Wei, and *Camellia japonica* (L.).

We used 35 temporary sample plots data of *Phyllostachys edulis* (moso bamboo) forests in Yixing Forestry Farm, Jiangsu Province. Each sample plot was 20 × 20 m (**Figure 1**). In July 2022, a total of 1,374 bamboo samples were collected from 4 blocks and 35 sample plots (**Table 1**). The diameter at breast height (DBH), total height (H), HCB (the height from the ground to the base of the first normal green branch as a part of the crown, excluding secondary branches, i.e., epicormic and adventitious), and CW in four perpendicular directions (CW_S_, south; CW_N_, north; CW_W_, west; CW_E_, east) of all living bamboos with a DBH > 5 cm were measured. Each component of the bamboo crown was measured as the horizontal distance from the center of the bamboo stem to the maximum range of the bamboo crown (Marshall et al., 2003). The CW was measured using a horizontal rangefinder and the average CW was calculated as follows: (CW_S_ + CW_N_ + CW_E_ + CW_W_)/2. DBH measurements were performed using a diameter tape, and an ultrasonic altimeter was used to measure H and HCB. The canopy density (CD) was measured using fisheye lenses. Because of the unique growth characteristics of moso bamboo forests with a vegetative cycle of 2 years (on- and off-year), stand age was expressed as “du” (Tang et al., 2016). One “du” (I) represents 1–2 years, and 2 and 3 “du” (II and III) correspond to 3–4 and 5–6 years, respectively (Tang et al., 2016). Based on the locations of the sample plots and the growth status of forests on slope and direction, we grouped the sample plots into four blocks according to their slopes (**Table 1**). Data from the bamboo individuals and stand factors investigated are summarized in **Table 2**.

**Table 1:** Division of bamboo forest area into four blocks and number of sample plots in each block.

Block	Slope (degree)	Slope direction	Slope position	Number of sample plots
1	0-3°	East-west	Downhill	9
2	15-20°	East-west	Mid slope	8
3	5-10°	East-west	Downhill	9
4	11-15°	South-north	Mid slope	9

**Table 2:** Summary measurements of bamboo forest characteristics.

Variable	Min	Max	Mean	Std
DBH (cm)	5.00	15.60	10.45	1.5757
H (m)	5.00	17.90	11.88	1.9079
HCB (m)	1.40	14.30	6.75	1.8888
A (du)	1	5	1.78	1.6087
BA (m^2^/ha)	11.32	35.15	25.70	7.4553
MDBH (cm)	9.361	11.752	10.435	0.5861
BAL (m^2^/ha)	0	56.16	23.44	14.1470
QMD (cm)	9.512	11.825	10.543	0.5723
N (culm/ha)	1200	3750	2939	783.0678
CD	0.4	0.8	0.6292	0.1631
RD	0.4406	1.4384	0.9913	0.1402
CW (m)	2.027	4.862	3.091	0.3845

Min, minimum; Max, maximum; Mean, average value; Std, standard deviation; DBH, diameter at breast height; H, bamboo height; A, age; HCB, height to crown base; BA, base area; MDBH, Arithmetic mean diameter at breast height; QMD, quadratic mean DBH; CD, canopy density; BAL, total basal area of all bamboos with diameter larger than that of the subject bamboo; N, Number of culms per hectare; RD, elative diameter (ratio of DBH of individual to QMD); CW, crown width.

### 2.2 CW models

#### 2.2.1 Base function

In bamboo forests, DBH comprises an important factor reflecting the growth of bamboo forests. DBH measurements are accurate and represent a relatively reliable variable in bamboo forest surveys. Our analysis started by fitting the eight commonly used forest growth functions (**Table 3**) to the data and choosing the best performing function based on major statistical indicators (Eqs. 9–12). The best fit function was then used for further extension and evaluation.

**Table 3:** Form of the base functions. CW_*ijk*_ is the crown width of the *k* bamboo in the *j* sample plot in the *i* block, DBH is the diameter at breast height of the *k* bamboo in the *j* sample plot in the *i* block, *β*_1_−*β*_3_ : model parameters.

Specification of function	Function form	Source	Eq.
*CW*_*ijk*_=*β*_1_+*β*_2_ *DBH*_*ijk*_	Linear	Sánchez-González et al., 2007; Sönmez, 2009	(1)
$CW_{ijk}=\beta_{1}DBH_{i_{jk}}^{\beta_{2}}$	Power	Sánchez-González et al., 2007; Sönmez, 2009	(2)
*CW*_*ijk*_=*β*_1_[1−*exp*(−*β*_2_ *DBH*_*ijk*_)]	Monomolecular	Sánchez-González et al., 2007; Sönmez, 2009	(3)
*CW*_*ijk*_=[*D B H*_*i j k*_/(*β*_1_+*β*_2_ *DBH*_*ijk*_)]^2^	Hossfeld 1	Sánchez-González et al., 2007; Sönmez, 2009	(4)
*CW*_*ijk*_=*exp*(*β*_1_+*β*_2_ *DBH*_*ijk*_)	Growth	Sönmez, 2009	(5)
*CW*_*ijk*_=*β*_1_ *exp*(*β*_2_ *DBH*_*ijk*_)	Exponential	Sönmez, 2009	(6)
*CW*_*ijk*_=*β*_1_[1−*exp*(−*β*_2_ *DBH*_*ijk*_)]^*β*_3_^	Richards	Fu and Sun, 2013	(7)
*CW*_*ijk*_=*β*_1_/[1+*β*_2_ *exp*(−*β*_3_ *DBH*_*ijk*_)]	Logistic	Fu and Sun, 2013	(8)

Four statistical indicators (MD, mean residual error; R^2^, coefficient of determination; TRE, total relative error; and RMSE, root mean square error; Eqs. 9–12), commonly used to evaluate the fitting behavior of models, were considered.


(9) $$MD=\frac{1}{n}\sum_{i=1}^{n}\left(CW_{ijk}-{\hat{CW}}_{ijk}\right)$$

(10) $$R^{2}=1-\frac{\sum_{i=1}^{n}{\left(CW_{ijk}-{\hat{CW}}_{ijk}\right)}^{2}}{\sum_{i=1}^{n}{\left(CW_{ijk}-{\bar{CW}}_{ij}\right)}^{2}}$$

(11) $$TRE=\sum_{i=1}^{n}|CW_{ijk}-{\hat{CW}}_{ijk}|/\sum_{i=1}^{n}{\hat{CW}}_{ijk}$$

(12) $$RMSE=\sqrt{\frac{1}{n}\sum_{i=1}^{n}{\left(CW_{ijk}-{\hat{CW}}_{ijk}\right)}^{2}}$$

where *CW*_*ijk*_ and ${\hat{CW}}_{ijk}$ are the measured and predicted CW for the *k* bamboo in the *j* sample plot in the *i* block, respectively, and *n* represents the number of CW observations.

#### 2.2.2 Factors affecting CW

In addition to DBH, other factors may significantly influence CW and are related to stand variables, such as bamboo size and stand vitality, site, and competition factors (Sánchez-González et al., 2007; Sönmez, 2009; Fu et al., 2013). Thus, we evaluated the impact of 12 variables on CW: total height (H), HCB, stand density (N), CD, base area (BA) (bamboo size and stand vitality variables), arithmetic mean diameter at breast height (MDBH), quadratic mean diameter at breast height (QMD), the total base area of all bamboo with diameters larger than that of the tested bamboo (BAL), relative diameter (RD, ratio of individual DBH to DBH), slope, slope position, and humus thickness (site condition factor).

We used graphical analyses and relevant statistical tests to select significantly influencing variables (Uzoh and Oliver, 2008). RMSE (Eq. 12) and Akaike Information Criterion (AIC) were used to evaluate different combinations and logarithmic transformations of the variables and identify the significant variables. We used the R *nls* function for fitting the models and selected the optimal model, which was then used to construct a two-level NLME CW model.

#### 2.2.3 Two-level NLME CW model

In the optimal model selected above, random effects were introduced at the block and sample plot levels. The model with the smallest AIC and largest log likelihood was selected for further analysis (Yang et al., 2009). The likelihood ratio test was used to avoid over-parametrization (Fang and Bailey, 2001). We reduced the heteroscedasticity problem using the variance function in the NLME CW model. Three variance-stabilizing functions were evaluated: the exponential (Eq. 13), power (Eq. 14), and constant plus power functions (Eq. 15) (Davidian and Giltinan, 1995; Pinheiro and Bates, 2000). We included the best-performing model in the final model. We used the AIC and likelihood ratio test to select the best-performing function (Pinheiro and Bates, 2000; Fang and Bailey, 2001).


(13) $$Var(\xi_{ijk})=\sigma^{2}\exp(2\gamma MDBH_{ij})$$

(14) $$Var(\xi_{ijk})=\sigma^{2}MDBH_{i_{j}}^{2\gamma }$$

(15) $$Var(\xi_{ijk})=\sigma^{2}{\left(\gamma_{1}+MDBH_{i_{j}}^{2\gamma_{2}}\right)}^{2}$$

where *MDBH*_*ij*_ is the arithmetic mean diameter at breast height (MDBH) of the *j* sample plot in the *i* block and *γ*, *γ*_1_, and *γ*_2_ are parameters to be estimated. *σ* is the residual variance of the CW model.

### 2.3 Parameter estimation

All basic models were estimated using the R *nls* function, and the NLME CW model was estimated using the *nlme* function (Lindstrom and Bates, 1990; Pinheiro and Bates, 2000). The fitted model variants were evaluated using the major statistical indicators (Eq. 9–12).

### 2.4 Model prediction

The optimal NLME CW model selected above was used for predictions, with and without the random effects considered. The former and latter models are called the M-response model and localized model, respectively; the latter is response calibrated with the local measurements (before the measurement of a response variable, in our case, CW) (Calama and Montero, 2004; Yang et al., 2009). We applied the empirical best linear unbiased prediction (EBLUP) to estimate the random effects and response calibration of the NLME CW model.


$${\hat{u}}_{i}=\hat{\Psi }Z_{i}^{T}{\left({\hat{R}}_{i}+Z_{i}\hat{\Psi }Z_{i}^{T}\right)}^{-1}e_{i}$$

(16) $$=\hat{\Psi }Z_{i}^{T}{\left({\hat{R}}_{i}+Z_{i}\hat{\Psi }Z_{i}^{T}\right)}^{{-}^{1}}[y_{i}-f(\hat{\beta },u_{i}^{*},x_{i})+Z_{i}u_{i}^{*}]$$

Where ${\hat{u}}_{i}$ is the estimated random effect for the *i* sample plot (*i* = 1,…M); *f*(·) is the NLME CW model; $\hat{\beta }$ is the vector of the fixed effects *β* ; *x*_*i*_ is the vector of the predictor variables; $\hat{\Psi }$ $\hat{\Psi }$ is the estimated variance-covariance matrix for the random effects *u*_*i*_ (*i* = 1,… M); ${\hat{R}}_{i}$ is the estimated variance-covariance matrix of the errors *e*_*i*_ *e*_*i*_ ; and *Z*_*i*_ is the *f*(·) *n*_*i*_×*q* design matrix of the partial derivatives of the estimated NLME CW model *f*(·) *u*_*i*_ with respect to the random effects *u*_*i*_ . The EBLUP theory was applied to estimate random effects (Meng and Huang, 2009; Fu et al., 2017a).

### 2.5 Prior measurement strategy

The mixed-effects model estimates the random effects and response calibration using single or multiple samples measured in each sample plot. Various sampling methods affect the random effects differently, thereby affecting the prediction accuracy of the NLME CW model. A few studies have evaluated the number of sample trees necessary to predict the random effects of NLME CW models and suggest the optimal numbers (Fu et al., 2013; Fu et al., 2015; Fu et al., 2017a). However, based on our broad literature review, no study of a similar type has been conducted on bamboo forests. Therefore, to evaluate the variability of bamboo CW, we used the following sampling methods and identified the optimal strategy:

(1) 1–8 randomly selected bamboo(s) per sample plot;

(2) 1–8 bamboo(s) with the largest DBH per sample plot;

(3) 1–8 bamboo(s) with average DBH per sample plot, and

(4) 1–8 bamboo(s) with the smallest DBH per sample plot.

The RMSE and TRE statistics were used to evaluate the prediction performance of the various sampling methods, and each sampling method was repeated 100 times to obtain the average statistics.

We used a three-step iterative algorithm proposed by Meng and Huang (2009) that involves computing random effects at the block and sample plot levels to calibrate the two-level NLME CW model (Meng and Huang, 2009). The first step is obtaining the initial (approximate) prediction of the random effects. The second step is updating the estimation of the random effect values by adding the estimated values of the model fitting parameters. The third step is the repetition of Step 2 until the required accuracy is obtained using iteration *k*. The precision was $\left|{\hat{\mu }}_{i}^{k}-{\hat{\mu }}_{i}^{k-1}\right|<0.0000001$ and $\hat{\mu }$ is the estimated value of random effect). The computational process was implemented in R software (version 3.4.2) using the nlme function. Details on this process are available in modeling studies (e.g., Meng and Huang, 2009, Fu et al., 2017a).

### 2.6 Model evaluation

The effectiveness of the NLME CW model can be evaluated using an independent dataset. However, this data acquisition method would be laborious and limited. Therefore, this study employed leave-one-out cross-validation (LOOCV) to validate the CW model. One sample plot was removed each time, and the remaining sample plot data were used to fit the model. This step was repeated 35 times. Next, the average statistical indicators (Eqs. 9–12) were calculated using the difference between the predicted and observed CW values.

## 3 Results

We evaluated the model fitting performance using the LOOCV method. Model 8 (logistic form) showed relatively better fit statistics (**Table 4**) (Eq. 17). Therefore, Model 8 was selected for further analysis, i.e., for developing a mixed-effects model.

**Table 4:** Fit indicators of the basic models (**Table 3**).

Model fitting					Model validation			
Model	MD	RMSE	R^2^	TRE	MD	RMSE	R^2^	TRE
1	-1.72e-06	0.2694	0.5094	0.7531	-0.0004	0.2706	0.5048	5.6125
2	-0.0002	0.2669	0.5184	0.7392	-0.0005	0.2680	0.5141	5.5432
3	-0.0004	0.2644	0.5276	0.7251	-0.0005	0.2654	0.5234	5.4933
4	-7.08e-05	0.2644	0.5275	0.7252	-0.0001	0.2654	0.5236	5.4865
5	-0.0005	0.2741	0.4923	0.7797	-0.0010	0.2754	0.4868	5.8762
6	-0.0005	0.2741	0.4923	0.7797	-0.0010	0.2754	0.4868	5.8762
7	0.0001	0.2634	0.5310	0.7198	0.0002	0.2648	0.5257	5.5014
8	0.0002	0.2621	0.5356	0.7127	0.0003	0.2635	0.5305	5.4299

MD, mean residual error; RMSE, root mean square error; R^2^ coefficient of determination; and TRE, total relative error.


(17) $$CW_{ijk}=\beta_{1}/[1+\beta_{2}\exp(-\beta_{3}DBH_{ijk})]+\xi_{ij}$$

### 3.1 Extension of base model

As previously mentioned, only the variables that contributed significantly to CW variations were added to the optimal CW model (Eq. 18). These variables were H, HCB, and MDBH. This model performed satisfactorily (R^2^ = 0.5392, RMSE = 0.2611, and TRE = 0.7071).


(18) $$CW_{ijk}=\frac{\left(\beta_{1}+\beta_{2}MDBH_{ij}\right)}{\left[1+(\beta_{3}+\beta_{4}HCB_{ijk})e^{\left((\beta_{5}+\beta_{6}H_{ijk})DBH_{ijk}\right)}\right]}+\xi_{ijk}$$

where *β*_1_−*β*_6_ are the parameters to be estimated. *H*_*ijk*_ is the height of the *k* bamboo in the *j* sample plot in the *i* block; *MDBH*_*ij*_ is the arithmetic mean DBH of the *j* sample plot in the *i* block; *DBH*_*ijk*_ is the diameter breast height of the *k* bamboo in the *j* sample plot in the *i* block; and *CW*_*ijk*_ is the crown width of the *k* bamboo in the *j* sample plot in the *i* block.

**Figure 2:**
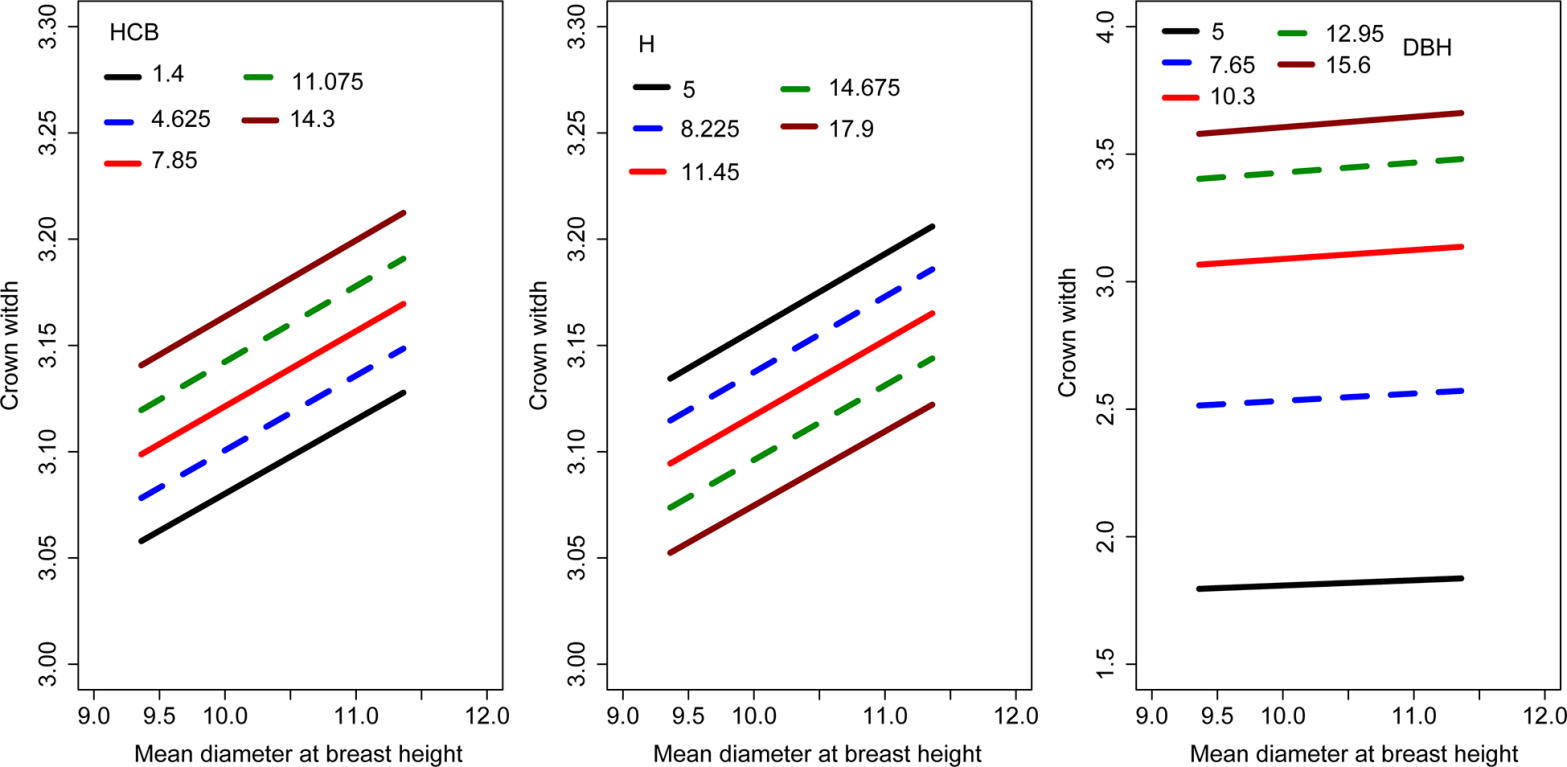
Effects of H, HCB and DBH on the CW. The curves were produced using the logistic model, i.e., Eq. 18.

We evaluated the effects of HCB, H, and DBH on CW using Eq. 18 *via* graphical simulation (**Figure 2**). As DBH and HCB increased, CW increased. However, CW decreased as H increased. DBH had the greatest influence on the variation in CW, followed by HCB and H.

**Figure 3:**
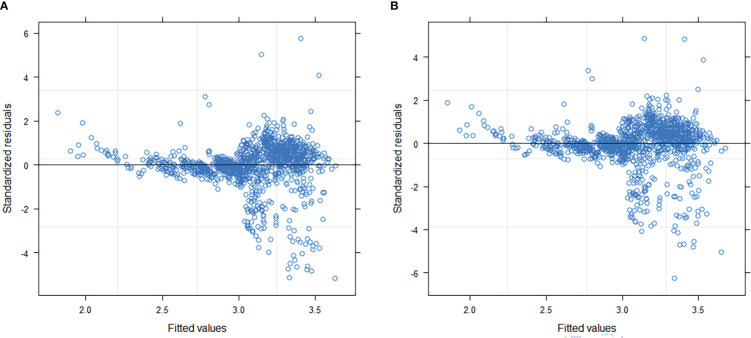
Residual distribution of Model 19 **(A)** and Model 19 + Eq. 15 **(B)**.

By comparing AIC and log-likelihood values of different parameter forms with random effects, only b_1_ and b_2_ in Eq. 19 were suitable for introducing block- and sample-plot-level random effects, which resulted in Eq. 19. The model produced minimum AIC (AIC = 189.7019), maximum log-likelihood (LL = -84.8509), and other fit statistics (R^2^ = 0.5724, RMSE = 0.2515, TRE = 0.6560). The constant plus power variance function (Eq. 15) accounted for heteroscedasticity most effectively (**Tables 4**, **5**; **Figure 3**). The final NLME CW model is as follows:

**Table 5:** Comparison among three variance functions (Eqs. 13–15) of NLME CW model (LL, logliklihood; AIC, Akaike Information Criterion; and L-Ratio, log-likelihood ratio test).

Variance function	AIC	LL	L-Ratio	P
Not added	189.7019	-84.85093		
Power	154.1289	-66.06443	39.07836	< 0.0001
Exponential	152.6235	-65.31175	37.57299	< 0.0001
Constant plus power	148.3223	-62.16113	45.3796	< 0.0001


(19) $$CW_{ijk}=\frac{\left[\beta_{1}+\mu_{i1}+(\beta_{2}+\mu_{i2}+\mu_{ij1})MDBH_{ij}\right]}{\left[1+(\beta_{3}+\beta_{4}HCB_{ijk})e^{\left((\beta_{5}+\beta_{6}H_{ijk})DBH_{ijk}\right)}\right]}+\xi_{ijk}$$

where $\xi_{ijk}\sim N(0,R_{i}=\sigma^{2}G_{i}^{0.5}\Gamma_{i}G_{i}^{0.5}),G_{i}=diag(\sigma^{2}{\left(\gamma_{1}+MDBH_{i_{1}}^{2\gamma_{2}}\right)}^{2},\ldots ,\sigma^{2}{\left(\gamma_{1}+MDBH_{i_{n}}^{2\gamma_{2}}\right)}^{2})$ , *μ*_*i*1_, *μ*_*i*2_ is forest block-level random effect, and *μ*_*i j*1_ is sample plot-level random effect.

### 3.2 Parameter estimation

The parameter estimates of Eq. 18 using the *nls* function and those of Eq. 19 estimated using the *nlme* function were significantly different from zero (*p< 0.05*). The estimated optimal CW model of Eq. 18 is as follows:


(20) $$CW_{ijk}=\frac{\left(3.3385+0.0427MDBH_{ij}\right)}{\left[1+(5.2787-0.0583HCB_{ijk})e^{\left((-0.3151+0.0011H_{ijk})DBH_{ijk}\right)}\right]}+\xi_{ijk}$$

where *ξ*_*ijk*_~*N*(0,0.2616) .

The estimated NLME CW model is shown in Eq. 21:


(21) $$CW_{ijk}=\frac{\left[3.4166+\mu_{i1}+(0.0417+\mu_{i2}+\mu_{ij1})MDBH_{ij}\right]}{\left[1+(4.6803-0.0518HCB_{ijk})e^{\left((-0.2943+0.0012H_{ijk})DBH_{ijk}\right)}\right]}+\xi_{ijk}$$

where


$$\mu_{i}=\left[\begin{array}{c} \mu_{i1}\\ \mu_{i2} \end{array}\right]\sim N\left\{\left[\begin{array}{c} 0\\ 0 \end{array}\right],{\hat{\Psi }}_{1}=\left(\begin{array}{ll} 3.08e-28 & -0.949\\ -0.949 & 1.09e-29 \end{array}\right)\right\}$$

$$\mu_{ij}=\mu_{ij1}\sim N(0,5.13e-05)$$

$$\xi_{ijk}\sim N(0,R_{i}=4.70e-26G_{i}^{0.5}\Gamma_{i}G_{i}^{0.5})$$

$$G_{i}=diag(4.70e-26{\left(9.08e+11+MDBH_{i_{1}}^{22.24}\right)}^{2},\ldots ,4.70e-26{\left(9.08e+11+MDBH_{i_{1}}^{22.24}\right)}^{2})$$

$$\Gamma_{i}=I_{i}$$

### 3.3 Model prediction

**Figure 4:**
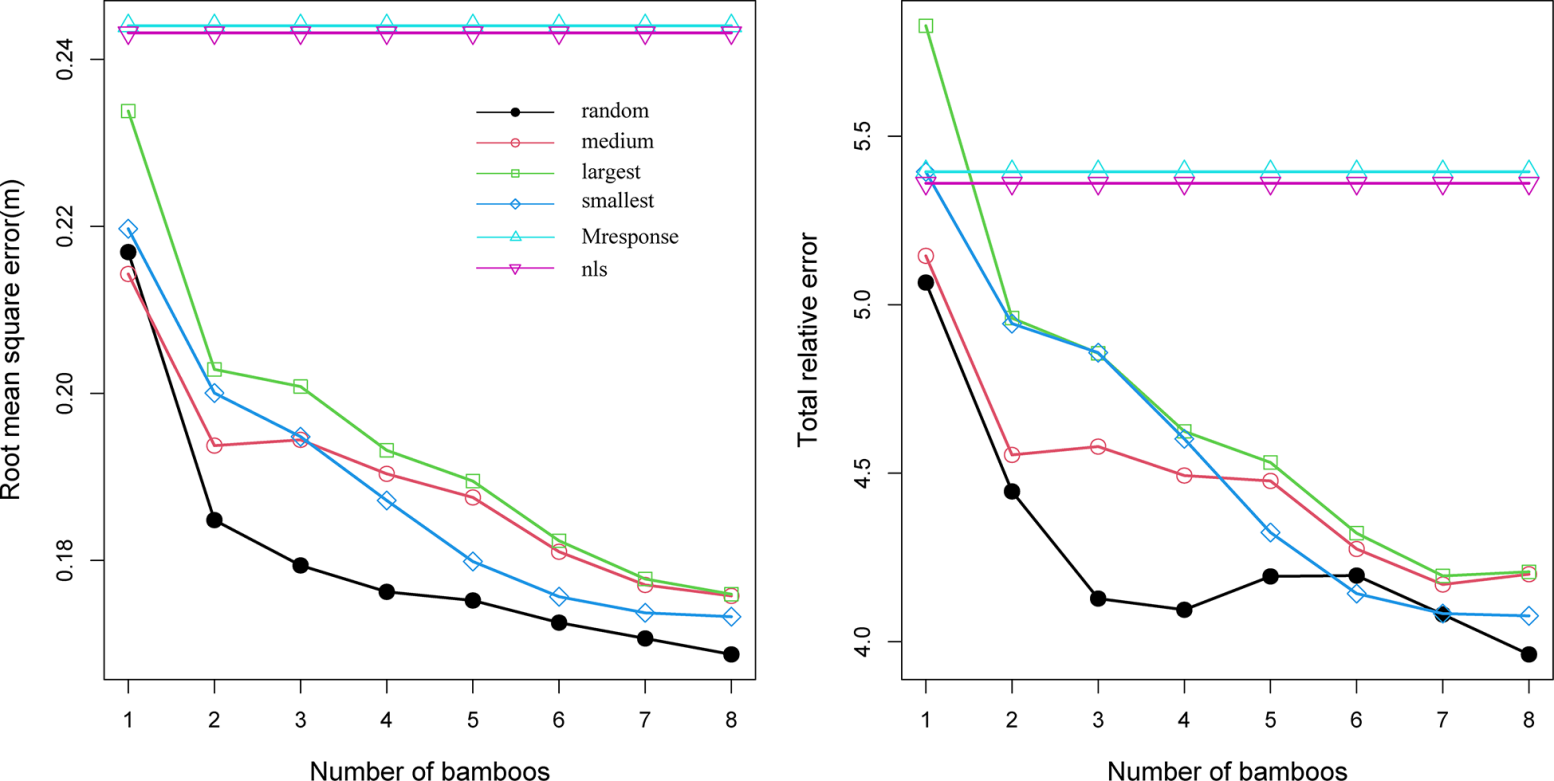
Root mean square error (RMSE) and total relative error (TRE) for the ordinary least squares (OLS) Model 18, Model 19 with the mean response (M response), and Model 18 with four sampling methods and sample sizes utilized within each sample plot, used to estimate the random effects (random: randomly selected DBH, largest: the largest DBH, medium: medium DBH, and smallest: the smallest DBH).

Four sampling methods were evaluated to predict the random effects of the NLME CW model (**Figure 4**). The RMSE and TRE of the four sampling methods exhibited the same trend: the prediction accuracy of the model gradually increased as the number of samples increased. When the minimum DBH was used, the model generated the minimum RMSE and TRE, indicating the suitability of the minimum DBH for calibrating and predicting bamboo CW. The decline rates of RMSE and TRE were the largest when the two bamboos with the smallest DBH were used for the random effects estimation.

### 3.4 Model evaluation

Using the LOOCV method, we evaluated the performance of the OLS CW, NLME CW, and M responses of the latter model. The prediction accuracy of the NLME CW model (Eq. 19) was higher than that of the other two model variants (Eq. 18, M-response of Eq. 19) (**Table 6**), thereby confirming that the block and sample plot level random effects were substantial.

**Table 6:** Statistical indicators of different forms of CW models using LOOCV methods (OLS-Eq.18; M-response-Eq.19; NLME-Eq.19).

Model(?)	MD	RMSE	R^2^	TRE
nls	-0.0028	0.2438	0.4082	5.3300
M response	-0.0012	0.2440	0.4075	5.3945
nlme	0.0001	0.2066	0.5724	4.7927

MD, mean residual error; RMSE, root mean square error; R^2^ coefficient of determination; TRE, total relative error.

## 4 Discussion

Research on the CW model has mainly focused on tree species and rarely on bamboo forests (Fu et al., 2017b; Fu and Sun, 2013; Fu et al., 2013; Fu et al., 2017a; Lei et al., 2018). Researchers select an appropriate model through the functional relationship between CW and DBH and then utilize the tree size vitality, site condition, and stand competition factors to develop their model. The factors used in most models reflect the site quality level (dominant height) (Fu and Sun, 2013; Fu et al., 2013; Lei et al., 2018). However, bamboos grow only in the first stage, after which their height does not change (Yen and Lee, 2011; Yen, 2016). Therefore, only using DH to reflect the site quality level is not appropriate. Therefore, in this study we considered the slope, slope direction, and slope location as the site quality levels of bamboo forests and used the mixed effect method to introduce the random effect of the group level to reflect the site quality level of bamboo forests. This method provides reliable technical support for CW calculations in bamboo forests.

Among the eight commonly used CW-DBH models evaluated in this study, the logistic form (i.e., Model 8) was used as the optimal basic model because it is superior to the other models, especially in flexibility. The logistic model is widely used to fit forest growth data, including DBH growth (Fu et al., 2018), survival (Buchman et al., 1983; Xie et al., 2022), mortality (Zhou et al., 2021b; Glover and Hool, 1979; Zhou et al., 2021a), tree CW (Fu and Sun, 2013; Fu et al., 2013; Lei et al., 2018), and HCB (Yang et al., 2020; Pan et al., 2020; Zhou et al., 2022). These examples show that the logistic model is sufficiently flexible in detailing potential tree growth variations, including bamboo CW variations.

Factors that potentially affect the crown should be considered (Davies and Pommerening, 2008; Uzoh and Oliver, 2008; Sönmez, 2009). We therefore evaluated the impact of 12 variables on bamboo CW. However, only HCB, H, MDBH, and DBH significantly affected CW. Thus, these factors included in modeling may significantly improve the model’s accuracy. Studies conducted on tree species have also found a positive correlation between CW and DBH and a negative correlation between CW and HCB (Fu et al., 2013; Sharma et al., 2016; Sharma et al., 2017; Ma et al., 2022). HCB and DBH are closely related to the size of the bamboo crown; thus, they may explain the significance of impacts on CW (Hasenauer and Monserud, 1996; Sharma et al., 2016; Zhou et al., 2022). MDBH describes the degree of bamboo crowding in a forest and explains the effects of competition on CW (Timilsina and Staudhammer, 2013). However, unlike the other studies on tree species (add relevant references here), CW was negatively correlated with H in this study. This may have resulted from the different growth characteristics of bamboo from those of other tree species, i.e., bamboo usually grow only within the first growth stage (Yen and Lee, 2011; Zhou et al., 2022). Bamboos with a smaller size were usually located in the lower layer of the stands with larger growth space and lower competition, which represents conditions most suitable for CW growth. Research has shown that relative spacing influences tree height and CW relationships (Saud et al., 2016; Pan et al., 2020). However, this study excluded this indicator because the stand density of trees will not change within a certain period. For bamboo, the annual number of bamboo shoots leads to changes in stand density, and bamboos are cut approximately 4–6 years after they emerge; thus, this index is not appropriate for use in modelling. Age is an important variable in bamboo forests. Bamboos of different ages have different abilities to compete for resources. Generally, bamboos in the mature or overmature stages are less competitive than those in the immature stages (Tang et al., 2016). In the study area, the bamboos were in the rapid growth stage, and few were larger than 3°, resulting in age having a minimal impact. Therefore, we replaced the age variable with MDBH and random effects in this study.

Various predictor variables affect CW differently. As DBH and HCB increased, CW increased, but it decreased with increasing H (**Figure 2**). In this study, the fitting effect did not change significantly after other variables were added to the model. Moreover, a model with too many variables and parameters may lead to over-parameterization and non-convergence (Fu et al., 2017b; Fu et al., 2017c; Fu et al., 2017a). In addition, including several stand or tree variables may increase the cost and time of an inventory. Therefore, determining an appropriate number of variables with the high accuracy required by forest managers would be difficult in forest modeling (Calama and Montero, 2004; Ademe et al., 2008). A simple model with a reliable prediction accuracy is therefore a desirable choice for effective forest management (Kiernan et al., 2008). Therefore, we retained only four variables in the final CW model (see Eq. 18), which are easily obtained from forestry databases.

The fitting accuracy of the model significantly improved after including the two level random effects. This is because the random effects parameters accounted for the difference in DBH changes in the different blocks within the bamboo forests and different sample plots within the blocks. The stand density of the studied bamboo forests ranged from 1,200 to 3,750 plants/ha, which largely affects CW variability (Essery et al., 2008). Because the effects of MDBH was assumed to be reacted in that of crowding degree or stand density, we did not consider introducing the number of bamboos per sample plot into the CW model.

To reduce inventory costs, the mixed-effects model estimates the random effects using as few samples as possible (Liu and Cela, 2008; Fu et al., 2017c). A small number of samples may capture the unobserved influence of stand variables on CW. Researchers have discussed the sample size necessary to predict random effects with reasonable accuracy (Fu et al., 2017b; Yang et al., 2020; Zhou et al., 2021a; Ma et al., 2022; Zhou et al., 2022). In this study, we evaluated the impact of four sampling methods on the prediction accuracy of the NLME CW model. Increasing the number of samples resulted in the statistical indicators (RMSE and TRE) showing a downward trend (**Figure 4**), which is more or less similar to results of studies on tree species (add relevant references here). A reasonably high prediction accuracy was achieved for the NLME CW model using the random effects estimated with only the smallest two bamboos per sample plot. Two bamboo poles were considered the optical number to predict the random effects of the NLME CW model from the perspective of the affordable sampling cost and prediction accuracy of the model. Similar empirical results have been reported in other modeling studies (Zhou et al., 2022).

The data used in this study were acquired from field surveys, and CW was calculated from four azimuth angles; this approach may lead to substantial errors in predicting CW and the crown area (Bragg, 2001; Marshall et al., 2003). These errors can be reduced by considering the geometric mean (Gregoire and Valentine, 1995). Because all the sample plots were distributed across pure bamboo forests, differences in the orientation of the crown diameter of bamboos in the sample plots were ignored. Therefore, the potential errors introduced by the arithmetic mean (CW) may be minimal and insignificant. Currently, remote sensing techniques are widely used in forestry research (García et al., 2010; Fu et al., 2018; Yang et al., 2020). Moreover, the factors influencing CW can also be estimated using remote sensing images, which can be largely supportive in constructing CW models and reducing investigation costs.

## 5 Conclusion

The nonlinear mixed effect CW model, which was developed using the DBH, HCB, arithmetic MDBH, bamboo height (H), and forest block- and sample plot-level random effects, demonstrated a promising precision level. Even with an increase in the number of samples, the prediction statistics gradually decreased, and the two bamboos with the smallest DBH per sample plot were used to calibrate the nonlinear mixed-effects CW model. This approach may therefore substantially reduce measurement costs and provide an acceptable accuracy. The model established in this study can be applied to bamboo CW predictions in forests similar to those in this study and may provide a basis for bamboo forest management.

## Data availability statement

The original contributions presented in the study are included in the article/supplementary material. Further inquiries can be directed to the corresponding author. 

## Author contributions

XZ, ZL, LL, and FG collected data; XZ, SF, and FG analyzed data; XZ, ZL, LL, RS, and FG wrote manuscript and contributed critically to improve the manuscript, and gave a final approval for publication.
